# Exploring New Frontiers: Alternative Breast Cancer Treatments Through Glycocalyx Research

**DOI:** 10.1155/tbj/9952727

**Published:** 2025-05-22

**Authors:** Ielizaveta Gorodetska, Anastasiia Samusieva, Tetiana Lahuta, Olga Ponomarova, Oleg Socha, Iryna Kozeretska

**Affiliations:** ^1^Institute of Radiooncology – OncoRay, Helmholtz-Zentrum Dresden-Rossendorf, Dresden, Germany; ^2^Department of Oncology, Shupyk National Healthcare University of Ukraine, Kyiv, Ukraine; ^3^Department of General and Molecular Pathophysiology, Bogomoletz Institute of Physiology, Kyiv, Ukraine; ^4^Department of Chemotherapy #1, Kyiv Municipal Clinical Oncological Center, Kyiv, Ukraine; ^5^Provincial Hospital. St. Padre Pio, Przemyśl, Poland; ^6^Taras Shevchenko National University of Kyiv, Kyiv, Ukraine

**Keywords:** alternative treatment, breast cancer, glycocalyx, immune system, molecular biology

## Abstract

Breast cancer (BC) treatment is developing toward more precise and personalized care through the approval of different comprehensive approaches. Clinical practice emphasizes significant patient-to-patient variability in treatment response among patients, even those with similar clinical and biological profiles. Recent studies have demonstrated that the glycocalyx is an essential organelle that plays an important role in many cellular processes and can be a promising target for treatment. The glycocalyx of cancer cells is a key component influencing the interaction between the tumor and the immune system. Glycan modifications attached to glycoproteins and glycolipids are a common characteristic of the transition to malignancy. We review how the specific structure and function of the glycocalyx are regulated at the molecular level, contribute to immune evasion, and can be overcome by using both traditional drugs and combination therapies, as well as drugs not previously used in standard cancer treatments, to address treatment resistance associated with glycocalyx alterations.

**Trial Registration:** ClinicalTrials.gov identifier: NCT00770354, NCT00925548, NCT01731587, NCT00088413, NCT00179309, NCT00986609, NCT05812326, NCT04020575, NCT05239143, NCT01279603, and NCT03562637

## 1. Introduction

Breast cancer (BC) is the most common cancer among women worldwide with over 2 million new cases reported in 2020, according to GLOBOCAN data. It also represents the leading cause of cancer-related deaths in women (Cancer Today). Ukraine mirrors these global trends, with 14,150 BC cases (14,036 women and 114 men) reported in 2021-2022 (NCR).

Significant advancements in BC management have been made in recent years. Today, the standards of BC treatment are based on determining the tumor's biological phenotype through analysis of established markers: estrogen receptor (ER), progesterone receptor (PR), human epidermal grows factor receptor 2 (HER2/neu), and marker of proliferation Kiel 67 (Ki67). ER is a biomarker expressed in approximately 70%–84% of BC cases. Its presence is associated with a less aggressive phenotype and better prognosis than ER-negative BC. PR is regulated by estrogen and plays a vital role in the development of the mammary gland. It is expressed in 75% of ER-positive BC cases [1]. HER2/neu is a transmembrane glycoprotein with tyrosine kinase activity, and HER2-positive tumors account for 15%–20% of BC cases [2]. The main treatment options for BC include loco-regional therapy (surgery and radiotherapy) and systemic therapy (chemotherapy, hormone therapy, anti-HER2 therapy, and immunotherapy). Hormone therapy is used for hormone-positive BC (ER^+^ and PR^+^), while anti-HER2 therapy is employed for HER2/neu-positive disease. Triple-negative breast cancer (TNBC) lacks targeted therapeutic options and remains an immense clinical challenge. Ki67 is a marker of cell proliferation, but its prognostic value is debated. While some studies have shown that high Ki67 levels are associated with a worse prognosis, others have found no clear evidence of its prognostic value [3].

Advances in molecular biology have enabled studying tumor characteristics in detail. The ONCOTYPE DX, MammaPrint, and other gene expression tests are widely used to predict recurrence risks, assess the likelihood of benefit from adjuvant chemotherapy, and provide a genomic risk score [4]. Current research is focused on identifying new predictive markers that can influence disease prognosis and response to systemic therapy, paving the way for individualized treatment approaches that improve disease-free and overall survival (OS).

The tumor microenvironment (TME), the ecosystem that surrounds a tumor inside the body, plays an important role in supporting cancer cell survival, local invasion, and metastatic dissemination [5]. It is also the site of direct interactions between tumor and immune cells. A key component influencing the tumor-immune system relationship is the glycocalyx [6]—a sugar-rich coating comprising glucosaminoglycans, glycoproteins, and glycolipids on the outer surface of the plasma membrane. Recently, the glycocalyx has emerged as a critical regulator of cellular homeostasis across various tissues. Alterations in the glycocalyx within BC have been identified as promising therapeutic targets.

The glycocalyx of tumor cell has recently been recognized as a significant driver of a cancer progression, underscoring its importance in developing new treatment options [7]. Tumorigenesis depends not only on oncogenic mutations but also a supportive microenvironment, often characterized by tissue stiffening due to alterations in glycocalyx [8]. Changes in the glycocalyx composition of cancer cells often contributes to treatment resistance by shielding tumors from immune attack and promoting cell survival. Despite advancements in immunotherapy, particularly with immune checkpoint inhibitors, the glycocalyx remains a powerful barrier to effective cancer treatment. Tumor cells exploit the glycocalyx to escape immune detection, highlighting the need for therapeutic strategies that target this protective layer [9].

This review explores the role of the glycocalyx in BC, focusing on its structure and function. In addition, it explores the potential of drug repurposing and combination therapies to overcome treatment resistance associated with glycocalyx alterations [10]. Of note, the role of the glycocalyx is being studied not only in BC but also in malignant neoplasms of other localizations, such as glioblastoma [11], bladder cancer [12], and pancreatic cancer [13]. In the next paragraphs, we review the roles of separate glycocalyx components in BC and discus the potential strategies for its targeting and further implication in diagnostics, prognosis, and treatment.

## 2. Glycocalyx

The glycocalyx, a meshwork of molecules surrounding animal cells, serves as an interface between the intracellular and extracellular environments. Secreted by the cell, it can be either membrane bound or exist independently [9, 14]. The glycocalyx contains various components, including free glycans, glycoproteins, proteoglycans, and glycolipids [9, 15]. Since these components are synthesized intracellularly before being assembled at the membrane, its composition is directly influenced by the cell's metabolic state, which is notably altered in cancer [14]. Thus, in most cases, tumor tissue is characterized by an increased size of the glycocalyx due to an increased concentration of proteoglycans and hyaluronic acid (HA), leading to tissue stiffening (when cells adhere to matrix proteins and apply tension, meeting resistance that reflects the stiffness of the tissue) [9, 16] and altered glycosylation [17]. Proteoglycans in particular contribute to extracellular matrix (ECM) stiffness and the tumor glycocalyx, including rearrangement of the collagen fibers [18, 19].

An important component of glycocalyx is sugars (glycans, also called polysaccharides), whose stoichiometry is extremely important for its structure. These sugars often undergo various chemical modifications, including oxidation of hedroxyl groups, N-acetylation, and sulfation [14]. Free glycans are typically polymerized, forming structures known as glycopolymers. When glycans are attached to lipids, the resulting molecules are called glycolipids. If 3 to 20 monosaccharides are attached to a protein, a glycoprotein is formed; when more monosaccharides are involved, it forms a proteoglycan [14]. The process of covalent attachment of glycans to molecules, typically proteins and lipids, is known as glycosylation. The donor molecules for the glycosylation are monosaccharides, and their availability is determined by cell's metabolic level [18, 20].

Proteoglycans are another major component of the glycocalyx. These diverse molecules consist of core protein (mainly members of syndecan (there are 4 types of this family in mammals) and glypican (there are 6 types of this family in mammals) and one or more covalently linked glycosaminoglycans (GAGs) chains. GAGs are linear polysaccharides composed of repeating disaccharide units that include an amino sugar [9, 16]. GAG chains can constitute the bulk of proteoglycans, which may also contain O- and N-glycans typically found on glycoproteins [21]. The expression of core proteins as proteoglycans, including syndecans and glypicans, differs between BC cells and normal cells [17]. For example, an increased proteolytic shedding of the extracellular domain of syndecan-1 (SDC-1), a type 1 transmembrane glycoprotein, is associated with increased metastasis [22]. This process is facilitated by heparanase, whose increased expression correlates with the progression of malignant processes, chemoresistance, and eventual disease relapse [23]. Heparan sulfate proteoglycans (HSPGs) are abnormally expressed in BC tissues: GPC1 is overexpressed, while GPC3 and GCP5 show reduced expression, and GPC4 expression is downregulated in metastatic breast tumors compared with nonmetastatic breast tumors [24, 25].

Glycoproteins are soluble membrane proteins characterized by the presence of oligosaccharide chains, which are always positioned on the outer surface of the cell membrane due to their hydrophilic properties [26]. Glycoproteins are formed through glycosylation, a complex and reversible enzymatic reaction [27]. Glycosylation can occur either as a posttranslational modification (PTM), or cotranslationally, as in the case with N-glycosylation [28]. Glycoproteins are classified into six types based on their glycosylation forms, with some glycoproteins bearing multiple glycosylation sites [27].

• N-glycosylation: oligosaccharides are attached to the nitrogen atom on an asparagine residue [29].
• O-glycosylation: oligosaccharides are attached to oxygen, typically on serine or threonine residues [30].
• C-glycosylation: a carbon–carbon bond links mannose to the indole ring of tryptophan [21].
• S-glycosylation: oligosaccharides are attached to the sulfur atom of cysteine residues [31].
• Phosphoglycosylation: phosphodiester bonds attach glycans to serine or threonine residues [32].
• Glypiation: a glycosylphosphatidylinositol anchor is added, linking a protein and phospholipid via a glycan core [33].

The glycan portion of glycoproteins consists of oligosaccharides containing approximately 3–20 monosaccharides [14].

The next group of glycocalyx molecules is glycolipids, which are complex molecules consisting of a ceramide lipid moiety linked to a glycan chain of variable length and structure. In human cells, glycolipids are represented exclusively by glycosphingolipids (GSLs). They are divided into several subclasses, including cerebrosides, which are single-glycosylated GSLs (Glc–Cer or Gal–Cer); globosides, which are polyglycosylated GSLs (Lac–Cer); and gangliosides, which contain at least one sialic acid attached to the Lac–Cer structure and mainly expressed in the outer layer of the plasma membrane in nearly all vertebrate cells [9]. Dysregulation of both the quantitative and qualitative composition of glycolipids in the glycocalyx has been observed in BC [34].

The synthesis of glycoproteins and glycolipids occurs within the cell, after which the molecules are transported to the ECM. GAGs are categorized into four main groups based on the repeating disaccharide units they contain: heparin/heparan sulfate, chondroitin sulfate/dermatan sulfate, keratan sulfate, and hyaluronan [35] or heparin (HP), heparan sulfate (HS), dermatan sulfate (DS), chondroitin sulfate (CS), keratan sulfate (KS), and HA [36]. GAGs can play an important role in naming of proteoglycans; for example, if a core protein contains the GAG heparin sulfate, it is referred to as a heparin sulfate proteoglycan (HSPG). These proteoglycans can either be anchored to the cell surface or released into the ECM. The GSL Globo H is expressed on the surface of numerous tumor cells including BC cells and growing evidence suggests that these GSLs can modulate the immune response to tumor cells [37]. Interestingly, it is expressed exclusively on tumor cells and is absent in the glycocalyx of normal cells [38].

In previous years, the functions of the glycocalyx were limited to a barrier between the cell and the ECM. However, recent studies have shown a significant diversity and importance of its functions, leading to its recognition in the literature as an organelle [14]. The glycocalyx continues to be considered as a physical barrier, protecting the cell from pathogens and nanoparticles [39, 40]. In addition, it plays an essential role in cell signaling [41]. The endothelial glycocalyx, which is in constant contact with the bloodstream, acts as a vital mechanosensor on endothelial cells [42]. This organelle plays a pivotal important role in cell adhesion, migration, and mostly any cell-surface interactions [9]. Another critical function of the glycocalyx in tumorigenesis is its role in managing cell morphology [14].

In recent years, there has been a surge in research focused on targeting the glycocalyx as a potential therapeutic modality. A study of Ščupáková and co-authors investigated N-glycosylation changes in 17 metastatic BC patients. The authors found that N-glycan abundance increased during metastatic progression, highlighting the clinical importance of specific glycans as potential diagnostic markers and therapeutic targets [43]. One recent study showed that VEGF alters the glycocalyx of BC cells, enhancing their adhesion and transmigration across the blood–brain barrier. This suggests that targeting glycocalyx dynamics could be a therapeutic strategy for preventing brain metastasis [44].

Furthermore, we will meticulously investigate the roles of individual glycocalyx components in BC progression, metastasis, and therapeutic resistance, with the aim of identifying novel avenues for improved treatment strategies.

## 3. Glycoproteins

The majority of glycoproteins in the glycocalyx are represented by mucins, which form a gel-like membrane on the cell surface and modulate intercellular communication [9]. In addition, other critical components include P-selectin glycoprotein ligand-1 (PSGL-1), a critical regulator of selectin binding to the endothelial surface [45]; CD43 (leukosialin or sialophorin), which acts as a selectin-dependent trafficking receptor [46]; and CD44, a glycoprotein that binds to its principal ligand, hyaluronan (HA), as well as collagen and fibronectin in the ECM [47]. The protein backbone of mucins is composed of tandem repeats of specific nucleotide sequences known as mucin domains, with each domain capable of being glycosylated. In the glycocalyx, the vast majority of glycosylated molecules are mucins [14].

Approximately half of all human proteins are glycosylated, and a major part of Food and Drug Administration (FDA)-approved cancer biomarkers are glycoproteins or carbohydrate antigens [48]. Glycoproteins are ideal biomarkers because they enter circulation from tissues or blood cells through active secretion or leakage, making them assessable for analysis in serum [49].

### 3.1. CA 15-3

For the BC, CA 15-3 (also known as MUC1) is a well-documented glycoprotein biomarker [50]. MUC1 is a large transmembrane glycoprotein containing of three main domains: a large extracellular region, a membrane-spanning sequence, and a cytoplasmic domain [51]. The MUC1 gene is located on the long arm (q) of chromosome 1 at position 21, a region frequently altered in BC cells [51]. Overexpression of MUC1 in cancer is caused by increased gene dosage and transcriptional levels, as well as a loss of posttranscriptional regulation. Studies on epigenetic regulation have shown that methylation of histone H3-K9 and the CpG islands in the MUC1 promoter lead to transcriptional repression [52]. MUC1 expression is also regulated post-transcriptionally. MUC1 mRNA contains the seed sequence for microRNA (miR)-125b in the 3′ untranslated region (UTR) and the loss of miR-125b expression in BC cells contributes to MUC1 overexpression [53].

In humans, multiple isoforms of MUC1 arise from alternative splicing, exon skipping, and intron retention. A recent study identified 78 different isoforms of MUC1 [54], with MUC1/Y isoform being highly expressed in breast cells [55]. Tumor-associated MUC1 differs from the form expressed in normal cells, both in its biochemical properties and its cellular distribution. Normally expressed MUC1 contains extensively branched Core 2 O-glycans, whereas MUC1 in BC cells predominantly exhibits Core 1 O-glycans [56]. This difference is largely due to the fact that tumor-associated MUC1 is highly sialylated [57].

The function of MUC1 in BC development has been extensively studied using preclinical mouse models. Muc1^−/−^ mice, which express high levels of polyomavirus middle T antigen in the mammary gland, able to develop BC but show a substantial delay in disease progression and metastasis compared to Muc1^+/+^ mice [58]. MUC1 mediates the proliferation of BC cells by stimulating the production of growth factors such as platelet-derived growth factor A (PDGF-A), PDGF-B and connective tissue growth factor (CTGF), which activate the MAPK and PI3K/Akt pathways [59, 60]. MUC1 also physically interacts with HIF-1*α*, acting as a modulator of the hypoxic response by regulating its expression, stability, and activity [61]. MUC1 enhances CIN85-dependent BC cell migration and invasion *in vitro* [62]. MUC1 overexpression interferes with integrin-mediated cell adhesion to the ECM, increasing the invasiveness of cancer cells [63]. The altered glycosylation of MUC1 allows it to function as a ligand for cell adhesion molecules such as selectins and intercellular adhesion molecule-1 (I-CAMs), facilitating the adherence of MUC1-expressing circulating tumor cells (CTCs) to endothelial cells and aiding in the establishment of secondary tumors at distant sites [64]. MUC1-induced factors such as CTGF, PDGF-A, and HIF-1*α* not only stimulate angiogenesis but also promote the migratory and invasive properties of cancer cells [59, 65]. Furthermore, MUC1 overexpression contributes to chemoresistance and resistance to apoptosis by reducing intracellular reactive oxygen species (ROS) levels through the upregulation of superoxide dismutase, catalase, and glutathione peroxidase [65]. Furthermore, MUC1 increases resistance to chemotherapeutic drugs by upregulating multidrug resistance genes and proteins, particularly multidrug resistance protein 1 (MRP1) in pancreatic cancer [66].

Given the functions mentioned above, tumor-associated MUC1 represents a promising BC biomarker and therapeutic target. Jing and co-authors demonstrated that MUC1 overexpression is associated with poor prognosis in patients with BC and is linked to MUC1 promoter methylation status based on the analysis of multiple large publicly available databases [67]. The MUC1-N tandem repeats are highly immunogenic in mice, leading to the development of several monoclonal antibodies (MAbs) targeting this subunit, with some proceeding to clinical evaluation [68]. AS1402 is a humanized immunoglobulin (IgG1) MAb that binds to the MUC1-N tandem repeats, inducing antibody-dependent cellular cytotoxicity against MUC1-positive BC cells [69]. Following the Phase I trial, Phase II trial of AS1402 in combination with letrozole was conducted in patients with advanced or metastatic ER^+^ BC. However, the results showed that adding AS1402 to letrozole did not enhance its efficacy compared with letrozole alone [70]. Few anti-MUC1 vaccines have advanced to later-stage clinical trials for BC treatment. L-BLP25 (Stimuvax; EMD Serono) is a liposome-based vaccine incorporating a MUC1-N tandem repeat peptide. Although Stimuvax was initially developed for both breast and lung cancer, its development for BC was terminated, and it is currently in a phase III trial for unresectable stage III NSCLC. Another phase II clinical trial, ABCSG 34, evaluated the efficacy and safety of Stimuvax in combination with neoadjuvant standard-of-care treatment for early BC. The MUC-1 vaccine, when added to standard neoadjuvant systemic therapy, significantly improved the distant recurrence-free survival (DRFS) and OS [71].

Therapeutic cancer vaccines aim to target tumor-specific antigens, such as differentiation antigens, overexpressed proteins, and cancer-testis antigens, to stimulate an immune response against cancer cells [72]. Various MUC1 vaccines have been developed to induce specific immune responses. PANVAC-V (NSC #727026) is a recombinant vaccinia virus-based vaccine containing transgenes for MUC1, CEA, and three T-cell costimulatory molecules (B7.1, LFA-3, and ICAM-1) [73]. PANVAC-F (NSC #727027) is a similar vaccine created by inserting the same transgenes into a replication-defective fowlpox virus. In a study where 12 BC patients were vaccinated with a subcutaneous priming dose of PANVAC-V followed by boosting doses of PANVAC-F, four patients achieved stable disease and one patient had a complete response according to Response Evaluation Criteria in Solid Tumors (RECIST) and remained on study for 37 months [74]. In another clinical trial, a combination of docetaxel and recombinant vaccine PANVAC-V, followed by boosting doses of PANVAC-F was evaluated. This clinical trial has demonstrated that the combination of docetaxel and the recombinant viral vaccine was superior to either agent alone in reducing tumor burden; and it enhances antigen-specific T-cell responses to antigen in the vaccine antigens as well as to cascade antigens derived from the tumor [75]. A study conducted at Case Medical Center (USA) evaluated the efficacy of MUC1 peptide-poly-ICLC adjuvant vaccine in boosting systemic immunity to MUC1 in women who had completed therapy for stage I–III triple-negative BC. Although this study was completed on January 21, 2016, the results have not published. Another exploratory clinical study aimed to assess the safety and preliminary efficacy of immunotherapy using PD-1 knockout anti-MUC1 CAR-T cells (AJMUC1) in treating advanced MUC1-positive BC. The study completion date was 16 November 2022, but the results have not yet been posted on ClinicalTrials.gov. As for today, several clinical trials are recruiting patients with different types and stages of BC. Another trial is a phase I/II study of adoptive immunotherapy for advanced MUC1∗ positive BC using autologous T cells designed to express either a chimeric antigen receptor (CAR), huMNC2-CAR44, or huMNC2-CAR22, which are specific for a cleaved form of MUC1 (MUC1∗). This trial is a Phase 1, open-label, dose-escalation, and expanded cohort study of P-MUC1C-ALLO1 in adult subjects with advanced or metastatic epithelial-derived solid tumors, including breast, ovarian, NSCLC, and colorectal cancer.

In a study by Daimon and co-authors, a small molecules screening was conducted to target MUC1-C-dependent CSCs, leading to the identification of salinomycin (SAL) an inducer of ferroptosis, as a potent inhibitor of MUC1-C signaling. They demonstrated that SAL suppresses MUC1-C expression by disrupting a NF-*κ*B/MUC1-C autoinductive circuit, which is necessary for ferroptosis resistance [76].

Sialylyransferases are enzymes involved in the biosynthesis of mucins. A recent study by Pucci and colleagues identified 17 glycosyltransferases associated with poor prognosis in BC, with ST6 N-acetylgalactosaminide alpha-2,6-sialyltransferase 4 (*ST6GALNAC4*) emerging as one of the top candidates [77]. Overexpression of this gene led to the formation of the sialyl-Tn (sTN) antigen, while knockdown of ST6GALNAC4 reduced metastasis by 95% *in vivo* [78, 79]. ST6GalNAc4 has been shown to promote cancer cell proliferation, migration, and invasion [80]. Although the exact mechanisms by which ST6GalNAc4 contributes to BC progression are still under investigation, it is evident that this enzyme plays a significant role in cancer development and progression.

In BC, MUC1 interacts with sialoadhesin (Sn), a receptor occurring on most infiltrating macrophages, specifically binding the sialylated form of MUC1 [81]. Macrophages, key players in both adaptive and innate immune responses, are classified into two subpopulations: Type I: classically activated macrophages and Type II: alternatively activated macrophages. Type I macrophages produce proinflammatory cytokines such as TNF-*α* and inducible nitric oxide synthase (iNOS), while Type II macrophages secrete anti-inflammatory cytokines such as IL-10 and arginase-1. The presence of human MUC1 has been shown to induce Type I macrophages in mouse models of colitis-associated cancer (CAC), highlighting MUC1's inflammatory role and its involvement in tumor promotion and progression [82]. Macrophages expressing Siglec-9 interact with sialylated MUC1 on tumor cells, promoting a protumorigenic phenotype through the release of factors such as interleukin-6 and macrophage colony-stimulating factors [82, 83]. Thus, a reduction in the ability of sialic acids, which serve as donors for sialyltransferases, could potentially disrupt these interactions.

Directly targeting the cytoplasmic domain of MUC1-C, which contains a CQC motif essential for its dimerization and oncogenic function, presents another promising therapeutic option [84]. To this end, cell-penetrating peptides and small molecules have been developed to specifically target the CQC motif and block MUC1-C dimerization in BC cells [84, 85]. The first-in-class cell-penetrating MUC1-C peptide inhibitor, GO-201, binds directly to the MUC1-C CD at the CQC motif, preventing its localization to the nucleus. Treatment with GO-201 has been associated with growth arrest and the induction of late apoptotic/necrotic cell death [85]. In addition, GO-201 has been shown to disrupt the interaction between MUC1-C and NF-*κ*B RelA, leading to decreased expression of NF-κB target genes in BC cells [86, 87]. In immunocompromised mice bearing MCF-7 (ER^+^), ZR-75-1 (ER^+^), and MDA-MB-231 (triple negative) breast tumor xenografts, administration of GO-201 resulted in prolonged tumor regressions [85]. Building on these findings, a novel MUC1-C inhibitor, GO-203, is currently undergoing Phase I clinical trials for patients with refractory solid tumors. The development of agents that block MUC1-C dimerization has further validated MUC1-C as a druggable target. Given the significant role of MUC1-C in BC progression, it is possible that this glycoprotein also contributes to tumorigenesis in other solid cancers, potentially offering a broad therapeutic target.

Two studies have demonstrated that synthetic glycopolymers, which truncate either MUC1 or MUC16, can reduce the glycocalyx density, thus slowing cancer development. This is achieved by mechanically enhancing cell-surface receptor function [88], which responds to mechanical stimuli and/or promotes the expansion of disseminated tumor cells *in vivo*. The latter effect is facilitated by fostering integrin adhesion assembly, allowing G1 cell cycle progression [89].

Mucin O-glycan degradation by mucinolytic bacteria offers an alternative strategy for reducing mucin levels, with potential implications for host–microbe interactions. One study focused on a glycoside hydrolase family 20 sulfoglycosidase (BbhII) from *Bifidobacterium bifidum*, which releases N-acetylglucosamine-6-sulfate from sulfated mucins. The authors showed that sulfoglycosidases play a role in mucin O-glycan breakdown *in vivo* and that the released N-acetylglucosamine-6-sulfate may affect gut microbial metabolism [90].

### 3.2. CA 27-29

CA 27-29 is a tumor marker primarily used in the management of BC. It is a soluble form of the glycoprotein MUC1 which is overexpressed in glandular epithelial cells, including breast tumors. The FDA has approved CA 27-29 for monitoring disease activity in BC patients, particularly for detecting recurrent or metastatic disease [91]. Recent advancements include the development of ultrasensitive electrochemical immunosensors for CA 27-29 detection, which show high specificity and sensitivity, making them valuable for early diagnosis and clinical treatment [92]. These sensors utilize nanocomposites such as Au/MoS2/rGO to detect CA 27-29 with high specificity and sensitivity. The immunosensor operates by monitoring the electrocatalytic current response of hydrogen peroxide reduction, which occurs through the immunoreaction between anti-CA 27-29 and the CA 27-29 antigen [92]. In addition, CA 27-29 is used in multigene assays to predict BC heterogeneity and recurrence scores, directing specific treatment strategies. These assays combine clinic-pathological information with genomic-imprint analysis to provide a comprehensive risk profile [93]. However, the clinical advantage of CA 27-29 is sometimes debated. For instance, it has been found to be less sensitive than CA 15-3 in certain settings, such as in monitoring metastatic BC [94]. In comparative studies, CA 27-29 has shown similar sensitivity and specificity to CA 15-3 in BC patients, but its levels can also be elevated in benign conditions, leading to potential false positive results. Combining CA 27-29 with other markers like CA 15-3 can improve diagnostic accuracy and monitoring of therapeutic responses [95, 96].

### 3.3. PSGL-1

PSGL-1 is a transmembrane glycoprotein primarily known for its role in leukocyte adhesion and migration. PSGL-1 is involved in immune cell trafficking and interactions with cancer cells. PSGL-1 expression can influence the immune response in the tumor microenvironment and contribute to cancer progression [97]. Research indicates that PSGL-1 expression is elevated in BC compared with normal tissue, with a strong correlation to tumor aggressiveness and metastasis in invasive ductal breast carcinoma [98]. While the exact mechanisms by which PSGL-1 contributes to BC progression are still under investigation, targeting PSGL-1 represents a potential therapeutic strategy.

### 3.4. CD43

CD43, also known as sialophorin, is a transmembrane glycoprotein typically expressed on hematopoietic cells, but its aberrant expression has been observed in various solid tumors, including BC. One study revealed that sialophorin expression patterns within BC cells can assist in classifying tumors into distinct subtypes, suggesting potential therapeutic targets [99]. Moreover, sialophorin may interfere with immune cell function, contributing to immune escape [100].

### 3.5. CD44

CD44 is another glycoprotein that is highly expressed in BC, and its overexpression is associated with poor prognosis [101]. CD44 is recognized as a marker of tumor-initiating cells across various tumors, including BC [101, 102]. Among its multiple isoforms, CD44v6 is particularly associated with cancer metastasis [103, 104]. Marangoni and colleagues showed that antibody-mediated CD44-targeting in human BC xenografts (HBCx) significantly reduces tumor growth, an effect attributed to the induction of growth-inhibiting factors [105]. Hu et al. demonstrated that miR-193b-5p acts as a tumor-suppressive miRNA by targeting CD44v6 in BC [106]. Resveratrol, a polyphenol found in grapes and red wine, has been shown to downregulate CD44 expression in BC cells, potentially reducing tumor cell adhesion, migration, and invasion [107]. In addition, resveratrol has been found to chemosensitize adriamycin-resistant BC cells by modulating miR-122-5p, which regulates key antiapoptotic proteins and cyclin-dependent kinases, leading to cell cycle arrest and increased apoptosis [108]. In another study, a series of nanoparticles composed of doxorubicin and tyrosine kinase inhibitors, loaded onto HA-coated dendritic polymers (HDDT nanoparticles), were used to target CD44 to stimulate immune response [109]. The results indicated that internalization of HA-dendrimer (Den)-DOX-apatinib (Apa) (HDDA) nanobombs (NBs) HDDA NBs was related to the CD44 molecules on the cell surface [109]. Curcumin, a polyphenol derived from turmeric, has also been shown to decrease the expression of CD44, a key marker of cancer stem cells. Curcumin reduces the proportion of CD44^+^/CD24^+^ cells, which are considered cancer stem-like cells, in BC cell lines [110].

## 4. Glycolipids

As previously mentioned, GSLs consist of a sphingosine backbone and are divided into several subclasses, including cerebrosides, which are singly glycosylated GSLs (Glc–Cer or Gal–Cer), globosides, which are poly-glycosylated GSLs (Lac–sCer), and gangliosides, which contain at least one sialic acid attached to the Lac–Cer structure [111, 112].

### 4.1. GSLs

GSLs have a significant role in organizing biological membranes, impacting membrane structure at different levels, as well as influencing the conformational and biological properties of membrane-associated proteins and multimolecular protein complexes. Alterations in the synthesis, functioning, and cleavage of GSLs in BC cells directly affect tumor microenvironment formation. Glycosyltransferases, the enzymes responsible for attaching sugar chains to lipids, function both within the cell and on the plasma membrane [113]. Iminosugars, carbohydrate analogs containing basic nitrogen atoms rather than oxygen in the sugar ring, act as potential glycosyltransferase inhibitors. The iminosugar lucerastat, an oral inhibitor, is currently in late-stage clinical development for treating diseases related to GSLs clustering [114]. It is important to emphasize that inhibiting GSL synthesis has been shown to improve insulin sensitivity and glycemic control in animal models of type 2 diabetes [115]. Several inhibitors of GSL synthesis are being studied mainly in cell models and may eventually be used in BC treatment [116].

GalCer serves as a precursor in the synthesis of sulfatides, which act as proapoptotic molecules. This process is mediated by GalCer sulfotransferase, and increased synthesis of sulfatide sensitizes BC cells to microenvironmental stressors, such as hypoxia and anticancer drugs [117]. Both GlcCer and Gal–Cer accumulation on the cell membrane contributes to drug resistance of tumor cells [118]. In MCF-7-AdrR BC cells, which express estrogen, progesterone, and glucocorticoid receptors, glucocerebroside levels were found to be 8–10 times higher than in normal cells [119]. Clinically relevant concentrations of tamoxifen, verapamil, and cyclosporin A, have been shown to significantly reduce glucosylceramide (GlcCer) levels in MCF-7-AdrR cells [120]. In addition, the overexpression of GlcCer synthase (UGCG, urine diphosphate-glucose ceramide glucosyltransferase) was significantly and selectively elevated in BC patients, particularly those with metastatic disease [121]. At the same time, the analysis of clinical samples revealed that high mRNA expression of UGCG is associated with longer survival time in BC patients [122], although the specific mechanism by which UGCG promotes BC cell proliferation and improve prognosis remains an unresolved. Phophatidylinositol-4-phosphate (PI4P) has been found to downregulate cellular GlcCer levels by inhibiting the interaction between GlcCer synthase and UDP-glucose [123]. GlcCers are precursors of globosides and gangliosides [116] and, therefore, their overexpression can contribute to an increase in these subclasses of glycolipids.

### 4.2. Cerebrosides

Cerebrosides, a type of GSL, are essential components of cell membranes. They consist of a ceramide backbone—a structure formed by a long-chain fatty acid attached to a sphingoid base—combined with a single monosaccharide, typically glucose or galactose [124]. It has been shown in MDA-MB-231 BC cells that accumulation of galactosylceramide (GalCer) inhibits apoptosis, allowing metastatic cells to survive in the aggressive microenvironment of tumor within target organs [125]. This accumulation of GalCer is associated with the high expression of GalCer synthase (UGT8, also known as urine diphosphate–galactose ceramide galactosyltransferase) in MDA-MB-231 BC cells. Notably, elevated UGT8 expression is specifically occurs in basal-like BC and serves as a predictor of poor prognosis in BC patients [126]. Zoledronic acid (ZA), a direct inhibitor of UGT8, suppresses the sulfatide biosynthetic pathway, thereby reducing BC cell migration and invasion [126].

### 4.3. Gangliosides

Sialic acids are an essential component of the glycocalyx. The attachment of sialic acids to sugar chains is the final step in the glycosylation of gangliosides [127] and increased sialylation is a hallmark of BC [128]. Sialidases, the family of glycosyltransferases, play a crucial role in the alterations of glycosylation patterns closely linked to cancer [129]. Overexpression of sialyltransferases leads to excessive sialylation, a common feature of cancer cells. Specifically, increased expression of ST3GAL1, ST3GAL2, ST3GAL3, ST6GAL1, ST6GAL2, ST6GALNAC2, ST8SIA1, ST8SIA4, and ST8SIA6-AS1 is associated with tumorigenesis, while ST6GALNAC2 is linked to the inhibition of tumorigenesis in BC [130]. However, the analysis of the BRCA cohort from the Cancer Genome Atlas revealed no significant increase in the mRNA expression of sialidase genes in BC, despite literature suggesting a correlation with a poor prognosis [77]. Most of these enzymes function intracellularly, although there is evidence that ST3GAL5 may also be expressed on the cell surface [113]. The decreased expression of ST8SIA1 correlates with reduced levels of GD3 and GM1 and its activity has been observed on the surface of macrophages [113]. Additionally, the sialidase that converts GM1 into GD3 is also expressed on the membrane surface [131]. Ganglioside degradation to ceramids can occur at the cell surface through the action of enzymes, such as sialidase neurominidarse3 (NEU3), *β*-galactosidase, and *β*-glucosidase [132]. NEU3, a key enzyme for ganglioside degradation, is significantly upregulated in human cancers, leading to apoptosis suppression [132]. SiRNA-mediated knockdown of NEU3 in HeLa cells induces apoptosis and increases GM3 synthase mRNA levels, while NEU3 overexpression has the opposite effect [133]. The roles of β-galactosidase and β-glucosidase in these processes in BC have yet to be investigated.

BC cells often exhibit higher levels of sialic acid on their surfaces compared with normal cells [133]. Several sialyltransferase inhibitors have been identified from natural products or microbial metabolites and from high-throughput screening methods. Most of these inhibitors can be broadly classified into (i) acceptor analogs, (ii) donor analogs, based on the structure of CMP-Neu5Ac, (iii) bisubstrate analogs, and (iv) transition-state analogs. In addition, some inhibitors are derived from natural sources [130]. Among the developed sialyltransferase inhibitors, compounds such as FCW34 and FCW66 have demonstrated efficacy in reducing BC cell migration, metastasis, and tumor growth by altering N-glycan sialylation and affecting key signaling pathways such as talin/integrin/FAK/paxillin and integrin/NF*κ*B [134]. FCW393, a derivative of lithocholic acid, selectively inhibits sialyltransferases ST6GAL1 and ST3GAL3, leading to reduced integrin sialylation and downregulating of proteins associated with cancer cell migration and invasion. This inhibitor has demonstrated significant antitumor and antimetastatic effects *in vivo* [130]. Inhibiting sialyltransferase ST3GAL1 can also reduce the sialylation of CD55, a complement regulatory protein, thereby increasing the sensitivity of BC cells to immune-mediated cytotoxicity [135]. Furthermore, soyasaponin I and its derivatives have been found to reduce alpha-2,3-sialyltransferase activity in a dose-dependent manner, leading to decreased alpha-2,3-sialic acid expression on the surface of BC cells [136, 137].

Ginsenosides, a class of natural steroid glycosides and triterpene saponins, are found almost exclusively in the *Panax ginseng* plant. It was demonstrated that ginsenosides block sialylation of *α*-2,3- and *α*-2,6-linked sialic acids in human liver cancer cell lines (HEPG2) and negatively interact with both ST3GAL1 and ST6GAL1 [137]. Although the mechanisms underlying the antineoplastic effects of ginsenosides on BC have not been fully elucidated, these compounds have been shown to be highly effective against BC both *in vitro* and *in vivo* [138].

*Sanguisorba officinalis*, commonly known as burnet, also exhibits neuraminidase inhibitory activity, reduced proliferation, induced S phase arrest, and triggers mitochondrial pathway apoptosis in BC cell lines MCF-7 and MDA-MB-231 [139, 140]. Similarly, screening the biological activities of *Crocus sativus* stigmas water extract—including antiallergic, antivirus, antineuraminidase, and anti-inflammatory effects—revealed its ability to inhibit neuraminidase activity by nearly 50% in TNBC MDA-MB-231 cells [141]. Alginate oligosaccharide, known for their antineoplastic activity, has been shown to exert their effects in prostate cancer by reducing sialylation [142]. Specifically, alginate oligosaccharides prepared by enzymatic hydrolysis (molecular weight: 1009 Da) and administered orally (10 mg daily) have been found to slow the progression of osteosarcoma [143]. In addition, He and colleagues reviewed alginate-based platforms for targeted drug delivery for BC [144].

Biotherapeutic molecules, known as antibody-enzyme conjugates, have the ability to selectively remove sialic acids from tumor cells, thereby modifying the glycocalyx and making the cells more accessible to natural killer (NK) cells. These conjugates show promise as targets for cancer immunotherapy [145]. Moreover, CAR T-cell engineered with Siglec-7 or Siglec-9 receptors, which bind sialic acid, showed a significant antitumor activity *in vitro* against several BC cell lines by targeting cancer-associated glycosylation patterns [146]. A recent study of Wang and colleagues highlighted the importance of the Siglec-15/sialic acid axis as a glycoimmune checkpoint in BC bone metastasis. Targeting this axis with anti-Siglec-15 antibodies shows promise for treating patients with BC that has metastasized to bone [147]. Another study found that collagen mineralization increases the thickness of the glycocalyx in BC cells, enhancing their resistance to immune attacks. This suggests that targeting glycocalyx sialylation could improve cancer immunotherapy outcomes [148].

Hypersialylation, characterized by an excess of negatively charged sialic acid on the cell surface, can arise from increased activity of sialyltransferases, decreased activity of neuraminidases, or a combination of both. Hypersialylation of the glycocalyx in metastatic BC cells promotes metastasis and immune evasion. One study investigates dietary interventions to metabolically target this hypersialylation, aiming to reduce metastatic potential of BC cells [149]. The results of this investigation remain to be elucidated.

Monosialo-gangliosides GM3 and GM1 have been described as suppressors of malignant properties of some cancer cells. However, in BC patients, the number of di-, tri-, and tetrasaccharide cores substituted by one or more sialic acid residues in the blood is higher compared to individuals without the disease [150]. Changes in the ratio of various gangliosides have been observed not only in the blood of patients but also on the surface of cancer cells [151].

Overexpression of B3GALT4, the glycosyltransferase responsible for ganglioside GM1 synthesis, has been shown to induce the epithelial–mesenchymal transition (EMT) process in MCF-10A cells [152]. At the same time, analysis of the BRCA cohort of the Cancer Genome Atlas showed that increased mRNA expression of the B3GALT4 gene is associated with a good prognosis in BC [77].

Disialo-gangliosides GD3 promotes tumor progression by affecting cell proliferation, adhesion and metastasis [153]. However, its accumulation in the cytosol can induce apoptosis, leading to mitochondrial damage in the early stages of apoptosis. Therefore, the effect of these gangliosides on neoplastic processes is determined by its localization. Disialo-gangliosides GD2, a specific type of glycolipid containing two sialic acid molecules, is actively expressed in BC stem cells, making it a potential tumor marker [151]. Interestingly, high expression of the enzyme responsible for synthesizing GD2 from GD3 has been shown to suppress the invasiveness of human BC MDA-MB231 cells [151, 153]. Cephalothin analogs have been found to inhibit GD3 synthase (GD3S), the enzyme involved in converting GD3 to GD2, in TNBC cells, thereby inhibiting tumor growth and metastasis [154]. Triptolide, a compound derived from the Chinese herb *Tripterygium wilfordii* Hook F, has demonstrated potent anticancer properties, particularly in BC. It has been shown to suppress the invasion, motility, and metastasis of BC cells through the inhibition of GD3S [155]. BMS-345541 (4(2′-aminoethyl)amino-1,8-dimethylimidazo(1,2-a)quinoxaline), a selective inhibitor of catalytic subunits of I kappa kinase (IKK) *α*/*β*, inhibits GD3S expression, mammosphere formation, and cell invasion activity *in vitro* [156]. BMS-345541 treatment has also been shown to reduce the tumor size and extended the survival time in breast tumor-bearing mice [157]. Furthermore, the anti-GD2 antibody dinutuximab has been found to inhibit the migration of triple negative stem-like BC cells [156, 157, 158].

### 4.4. Globosides

The globosides stage-specific embryonic antigen-3 (SSEA3), SSEA4, and Globo-H are highly and specifically expressed on the surface of BC stem cells [159]. The enzyme galactosyltransferase (*β*3GalT5), responsible for the biosynthesis of SSEA-3, is expressed in BC stem cells but not in normal cells. Knockdown of the gene encoding *β*3GalT5, which is involved in the Globo-series pathway, led to apoptosis in cancer cells specifically but had no effect on normal cells [159]. Globo-H vaccine has been shown to induce antibodies that target not only Globo-H, but also SSEA3 and SSEA4 [160]. In a Phase 3, randomized, open-label study, Adagloxad simolenin (Az-S), an anti-Globo H vaccine, demonstrated improved patient survival by 5–7 years [161].

## 5. Proteoglycans

Proteoglycans are complex molecules composed of a core protein with attached GAG chains. Expression levels and aberrant glycosylation of proteoglycans are key indicators of changes in the glycocalyx in BC cells. Heparanase, specifically HPSE, is responsible for cleaving HS chains within proteoglycans. This reaction is important for ECM remodeling and is linked to various pathological processes, including BC metastasis [162–164]. The HPSE exists in two isoforms: heparanase-1 (HPSE1) and heparanase-2 (HPSE2). Anticancer effects of HPSE inhibition are well documented. A wide range of HPSE inhibitors have been developed, including small-molecule inhibitors, synthetic and semisynthetic oligo- and polysaccharides (such as Necuparanib, Muparfostat, Pixatimod, Arixtra, and Aspen), neoproteoglycans, antibodies, and cyclophellitol-derived compounds. However, only a few of these compounds have advanced to clinical trials [157, 165]. *Curcuma wenyujiin* oil essential has been demonstrated to downregulate heparanase expression along with other anticancer effects in 4TI murine BC cells [166]. While there is substantial evidence supporting the anticancer activity of essential oils in BC, their application in medicine is limited due to their high volatility, low stability, and poor solubility. Recent advancements in nanotechnology are being explored to overcome these challenges [167].

### 5.1. Chondroitin Sulfate Proteoglycan 4 (CSPG4)

CSPG4 is a highly glycosylated transmembrane protein and a member of the chondroitin sulfate group of GAGs. Overexpression of CSPG4 has been associated with BC. CSPG4 is overexpressed in several cancer types, while its expression is typically restricted and low in normal tissues. CSPG4 contributes to tumor growth and progression by promoting angiogenesis, making it a promising target for BC therapy. Potential treatments include MAbs (various classic MAbs), CAR constructs based on MAbs, radioimmunotherapy [168], and recombinant SNAP-tag-based antibody-auristatin F drug conjugate [169].

It was shown that inhibition of mitogen-activated protein kinase (MAPK) by trametinib and GSK1120212 in human melanoma cell line M14 leads to the downregulation of CSPG4 expression [170]. In BC cells, the inhibition of MAPK/extracellular signal-regulated kinase (MEK/ERK) signaling pathway by U0126, a special MEK inhibitor, sensitizes cells to apoptosis [171]. Scutebarbatine A (SBT-A), a diterpenoid alkaloid, has a potent inhibitory effect on BC cells through modulation of MAPK signaling pathway [172]. While CSPG4 expression was not directly studied in the latter two publications, it is plausible to hypothesize that similar effects may occur in BC cells.

### 5.2. Lumican

Among small leucine-rich proteoglycans, lumican has been identified as having anticancer activity in invasive BC, including the inhibition of CD44 and HA synthases expression [173]. Low lumican (LUM) levels are associated with a worse prognosis in lymph node-negative invasive breast carcinomas [174]. Conversely, its upregulation is linked to poorer OS in patients with gastric cancer [175]. In addition, Guo et al. performed a meta-analysis of 19 BC datasets, involving 31 cohorts and indicated that LUM might be a prognostic risk factor, although the results were not statistically significant [176].

### 5.3. Syndecans

Syndecans act as a “signaling organizer” at the cell surface for other plasma membrane receptors, especially integrins and tyrosine kinases. Syndecan-1 (SDC-1) and Syndecan-4 (SDC-4), members of the HSPG family, are transmembrane molecules with SDC-1 also capable of containing chondroitin sulfate chains. High expression levels of both SDC-1 and SDC-4 have been observed in BC cells associating with aggressive cellular properties and poor prognosis [177, 178]. Treatment with Zoledronate or ZA, a class of bisphosphonates used to treat a number of bone diseases, has been shown to significantly downregulate SDC-1 and SDC-4 expression [179]. Nimesulide, a nonsteroidal anti-inflammatory drug, inhibits the tumorigenic activities of SDC-1 in primary effusion lymphoma, while methoxybenzamide derivative of nimesulide demonstrates potent anticancer activity with BC *in vitro* models [180]. In addition, SDC-4 expression is modulated by trastuzumab, a treatment used in BC treatment [178].

Synstatin, a peptide mimetic of the docking motifs in the syndecans, disrupts these interactions, thereby resulting in significantly decreased angiogenesis and tumorigenesis *in vivo* [181]. miR-135b-5p acts in the early prevention of BC metastasis by indirectly reducing SDC-1 expression [182]. Proteolytic cleavage of syndecan-1 promotes breast tumorigenesis, but compounds such as batimastat, benzo(*α*)pyrene, tranexamic acid, antithrombin III, and heparanase inhibitors can inhibit this process, potentially suppressing BC development [183].

### 5.4. Glypicans

Glypican-1 (GPC1), a member of the HSPG family, is overexpressed in BC cells and is associated with tumor progression, while its expression is low in normal tissues [25, 36]. Although there are reports on the use of anti-GPC1 antibody for certain forms of cancer, there are limited data regarding their efficacy in BC cells [184]. Moreover, GPC1-specific human and murine CAR T-cells have been developed. GPC3 expression is downregulated in BC cells, and its loss may contribute to cancer progression by inducing the activation of Hh signaling. Similarly, GPC4 expression is downregulated in metastatic breast tumors compared with nonmetastatic breast tumors. Thus, GPC3 and GPC4 act as a tumor suppressor [26]. The lncRNA GPC3 antisense transcript 1 (GPC3-AS1) is responsible for the upregulation of GPC3 in hepatocellular carcinoma cells, raising the question whether this mechanism could be leveraged to stimulate the expression of GPC3 in BC cells [185]. The reduced GPC5 and GPC6 expression has been observed in BC where it is associated with poor survival outcomes [25, 185].

## 6. Hyaluronan

Hyaluronan (HA), a linear GAG, does not form a proteoglycan. It is classified either as a component of ECM [186] or as a part of the glycocalyx [6]. Both perspectives hold merit, given HA's location and the mechanisms in which it is involved. HA is a principal ligand of the glycoprotein CD44 and also interacts with collagen and fibronectin in the ECM [47]. The protein receptor for hyaluronan-mediated motility (RHAMM) also binds to HA [186]. Hyaluronan is synthesized by three hyaluronan synthases (HASes) at the plasma membrane. Aggressive BC cells express high levels of HA synthase 2 (HAS2) and lower levels of HA synthase 3 (HAS3) compared to non-aggressive cancer cells [187]. Narvaez et al. reported that the overall frequency of genomic alterations in HAS2 was 27% in 1904 cases of breast invasive carcinoma [188]. High HAS2 expression significantly increases low molecular weight (LMW) HA in the glycocalyx of BC cells, which is considered a sign of poor prognosis. Enhanced hyaluronan synthesis is often accompanied by increased degradation due to the higher expression and activity of hyaluronidases (HYALs). The degradation of hyaluronan produces intratumoral hyaluronan fragments (LMW) that promote inflammation, angiogenesis, tumor growth, and metastasis [189]. Interestingly, while high molecular weight HA is associated with tissue homeostasis and protective effects, LMW HA is indicative of pathological conditions and plays a role in pro-oncogenic activities, driving local inflammation and tumor growth and invasion [190]. HA is localized in both the stroma and breast carcinoma cells, while hyaluronidase 2 (HYAL2) is predominantly localized in breast carcinoma cells [191]. HA was significantly correlated with cell proliferation and invasion ability and increased risk of recurrence, especially in the HYAL2-positive group. It seems that HA needs to be degraded by HYAL2 to exert protumorigenic effects, and comprehensive HA/HYAL2 status serves as a potent prognostic factor in BC. It was already demonstrated on melanoma and glioma that HYAL2 inhibitors prevent metastasis by inducing cell cycle arrest and apoptosis [192, 193].

The 4-methylumbelliferone (4-MU), an orally available dietary supplement and a drug approved in Europe (but not in the USA) for the treatment of biliary spasms, is a coumarin derivative mainly found in the plant family *Umbelliferae* or *Apiaceae*. An open-label, single-center, dose-response study of hymecromone in healthy adults showed that 4 days of oral 4-MU exposure led to a significant reduction in sputum HA levels, with both sputum and serum drug concentrations increasing in a dose-dependent manner. The most effective dose was 3600 mg/d [194]. The 4-MU specifically inhibits the HA synthesis by reducing the availability of HA substrates and inhibiting the activity of HAS2, leading to a reduction in cell proliferation and a decrease in pericellular matrix formation, which correlated with decreased invasiveness, providing a good strategy for inhibition of metastatic progression. The 4-MU binds to UDP-glucuronic acid, preventing its incorporation into HA, significantly reducing HA levels [195]. The 4-MU can also bind to glycolysis-associated proteins, such as Hsp90AA1, PGK2, GPD2, and GPI, potentially interrupting the energy supply to the tumor tissue. These glycolysis-associated proteins may be possible targets for cancer therapy [196]. The 4-MU has been shown to significantly suppress the incidence of metastasis and growth of CSCs in the bone could help to control tumor resistance. The low levels of HA and glucose in the tumor microenvironment could increase the sensitivity of BC cells to 4-MU treatment [197].

The 1,25-dihydroxyvitamin D_3_ (1,25D), the active form of vitamin D_3_, downregulates the hyaluronan synthase 2 (Has2) gene, thereby decreasing the hyaluronan production [198]. In cultures of murine mammary tumor cells treated simultaneously with 1,25D3 and 4-MU, the culture density was lowest compared to the culture that was exposed to only 1,25D3 [198]. Karalis et al. reported a new small molecule inhibitor of hyaluronan synthesis, the thymidine analog 5′-deoxy-5′-(1,3-diphenyl-2-imidazolidinyl)-thymidine (DDIT). This compound is more effective than 4-MU and displays significant antitumorigenic properties, inhibiting BC cell migration, invasion, proliferation, and cancer stem cell self-renewal capacities by suppressing HAS-synthesized hyaluronan [199]. The salicylate has been identified as a potent inhibitor of hyaluronan synthesis in BC cells. It activates 5′ AMP-activated protein kinase, which in turn inhibits hyaluronan production, possibly through the phosphorylation of HAS2, potently inhibited the migration and proliferation of TNBC cells [189].

Researchers are addressing the issue of elevated HAS2 activity in tumor cells through various approaches, including the use of long noncoding RNA HAS2-AS1, CRISPR/Cas9-mediated HAS2 knockout system, proteolysis targeting chimeric (PROTAC) technology [189]. Inhibition of HA secretion in cell culture by chitin synthesis inhibitors, particularly etoxazole, buprofezin, and triflumuron, suggests direct interaction with HAS2. More detailed studies of these interactions could provide new insights into modulating HA metabolism pathways in cancer [200]. The application of hyaluronidases to degrade HA in the tumor environment has also been investigated as a possible anticancer drug. Bacteriophage hyaluronidase has been shown to improve the bioavailability of anticancer medicines, and significantly reduce tumor size by 50% within 4 days in mice implanted with human BC cells following intravenous treatment [201]. Hyaluronidase 1 gene (HYAL1) is overexpressed in human BC, and its upregulation promotes cell growth, migration, invasion, and angiogenesis *in vitro* [202]. Targeting this enzyme might have positive effects in BC as well. However, this information should be taken with precaution since the article was retracted.

Corticosteroids have long been used in cancer therapy to provide pain relief, as serve as hormone therapy, and stimulate appetite and wellbeing [203]. Dexamethasone, a glucocorticoid, has been demonstrated to inhibit tumor cells *in vitro* and *in vivo* [204]. Glucocorticoids may directly or indirectly affect HA synthesis [205]. Therefore, it is plausible that corticosteroid treatment could lead to a reduction in HA levels within the glycocalyx of affected cells. However, due to the numerous side effects associated with corticosteroids, their localized use for various types of cancer could be considered, although relevant data in the literature are currently lacking [206].

**Figure 1:**
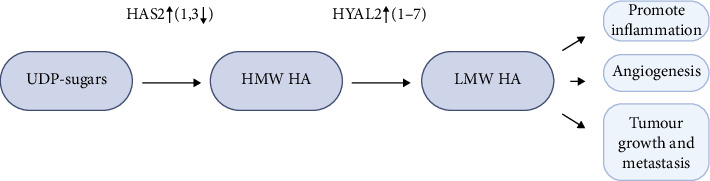
Schematic representation of the conversion of high molecular weight hyaluronic acid (HA) to low molecular weight HA (LMW-HA), a hallmark of pathological conditions that contributes to pro-oncogenic activities, driving local inflammation, tumor growth, and invasion.

Petasin (PT), derived from *Petasites japonicus*, is a highly potent mitochondrial electron transport chain complex I (ETCC1) inhibitor. It hinders glycosylation by reducing the concentration of HAS2 substrates, namely, UDP-sugars. PT treatment significantly inhibited lung and lymph node metastasis in the mouse metastatic mammary cancer spontaneous metastatic model (Jyg-MCB) [207, 208]. Other ETCC1 inhibitors, such as metformin and phenformin, are available but are considered insufficiently effective, while rotenone is highly toxic [208, 209]. The expression of HAS2 can also be reduced by Sirtuins 1, an NAD+-dependent deacetylase. It has been shown that this enzyme can be activated in human cell culture by resveratrol, an antioxidant polyphenol nonflavonoid compound and STR1720, selective synthetic [210]. Given its role in regulating HA synthesis, SIRT1 could potentially become a valuable target in BC therapy as well (Figure 1).

## 7. Mechanical Features: Tumor Stiffness

**Figure 2:**
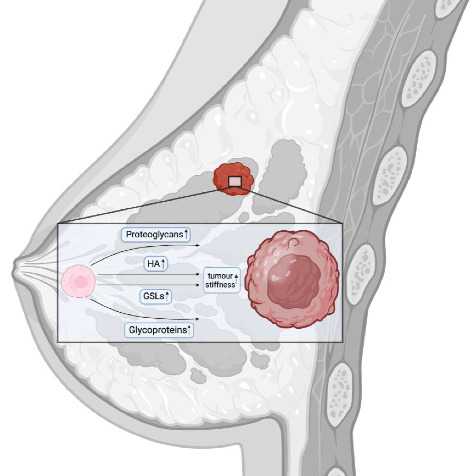
An increase in the level of proteoglycans, hyaluronic acid, glycosphingolipids and glycoproteins contribute to tissue stiffness.

Tissue stiffening is clinically correlated with tumor progression and aggressive adenocarcinoma of the breast [211]. Notably, even with the presence of oncogenic mutations and the loss of tumor suppressors, tumors may not develop without the microconditions that promote tissue stiffening [8]. Proteoglycans and hyaluronan in the glycocalyx of tumor cells play an important role in the formation of a stiffened BC, contributing to such processes as interstitial fluid pressure (IFP) or hydrostatic pressure; tensile force (TF) or stretching force, or solid stress; fluid shear stress (FSS) or shear pressure (Figure 2) [7, 212]. It has long been known that in patients with invasive ductal carcinomas, IFP was 29±3 (SE) mm Hg, compared with −0.3±0.1 mm Hg in those with normal breast parenchyma [213]. In addition, stiffened BC tissue is frequently accompanied by chronic inflammation, which further increases tissue stiffness [211]. The increased water retention may be mediated by an increase in the content of proteoglycans and hyaluronan in the glycocalyx [17]. Enzymatic degradation of hyaluronan has been shown to eliminate tumor swelling completely [214]. Therefore, reducing hyaluronan levels through treatments such as hymecromone [194] or, which decreases the concentration of the HAS2 substrate UDP-GlcNAc [188], as well as analogs of metformin or metformin, may help to reduce IFP in solid tumors. In addition, any factor that decreases the amount of proteoglycans in the glycocalyx of cancer cells could potentially reduce IFP (see section on proteoglycans).

Improving fluid drainage in cancer patients through herbal preparations with antitumor properties, such as Canephron—a traditional herbal medicine containing extracts of *Centaurium erythraea*, *Levisticum officinale*, and *Rosmarinus officinalis*—could also be considered as a strategy to reduce tumor swelling [215–217]. This drug has been effective in preventing genitourinary complications in BC patients undergoing chemotherapy and radiation therapy [218].

Inflammation is a key process in BC, and it is well-known that inflammation generally causes swelling at the site of occurrence [219]. Chronic inflammation can contribute to tissue stiffness. Numerous epidemiological and laboratory studies have shown that nonsteroidal anti-inflammatory drugs exert chemopreventive effects against BC [220]. Among the various inflammatory processes in BC, there is likely a mechanical component that contributes to tissue stiffness, and reducing inflammation may improve the availability of therapeutic drugs during BC treatment.

Solid stress, a mechanical abnormality in tumors, refers to the physical forces contained and transmitted by the solid components of the tumor. Reducing collagen and hyaluronan levels has been shown to decrease solid stress, decompress blood vessels, and enhance tumor oxygenation and drug delivery [221]. The antihypertensive drug losartan, a renin-angiotensin system inhibitor (RASI), reduces stromal collagen and hyaluronan production by downregulating profibrotic signals such as TGF-*β*1, CCN2, and ET-1, which are downstream of angiotensin-II-receptor-1 inhibition. Losartan has been shown to reduce solid stress in tumors, resulting in increased vascular perfusion and improved drug and oxygen delivery to tumors in breast and pancreatic cancer models [222]. The use of losartan increased the penetration of anticancer drugs into the tumor, which significantly increased the effectiveness of treatment [223, 224]. The long-term use of drugs of this group in hypertensive patients is associated with a reduced incidence of BC cases compared with those who do not use these drugs [225].

Tensile stress, or stretching, arises from local tissue due to ECM hardening, which in turn stimulates actomyosin contractility within cells via integrins [226]. Increased cell contractility is also characteristic of cells with bulky glycocalyx. A bulky glycocalyx has been shown to facilitate adhesion assembly and enhance integrin-mediated signaling, contributing to tensile stress development [88]. Consequently, reducing the thickness of glycocalyx by decreasing the levels of proteoglycans and hyaluronan may also reduce tensile stress in tumors.

Shear stress is caused by the flow of blood, and interstitial fluid cancer cells encounter as they extravasate into the vascular or lymphatic system during metastatic dissemination. These shear stresses can activate signaling pathways that increase tumor cell aggression and affect vascular permeability. It is hypothesized that the proteoglycan-rich glycocalyx of cancer tumors may contribute to this process [16]. The glycocalyx acts as a mechanosensory, responding to FSS by altering its composition and structure in several ways: (i) shear stress stimulates the production of glycocalyx components, such as HA, to reinforce the endothelial barrier; (ii) changes in glycocalyx composition and organization affect its permeability and biomechanical properties; and (iii) the glycocalyx transmits signals into the cell to initiate cellular responses [227–229]. However, among 1904 cases of breast invasive carcinoma, only 27% of the cases were found to have alterations in HAS2 [188]. This indicates that, while the role of HA in these processes requires further study, tissue stiffening is not solely determined by the influence of the glycocalyx. Understanding the influence of shear stress on the glycocalyx could inform drug delivery strategies by optimizing nanoparticle design.

## 8. Conclusions and Future Perspectives

Advancements in BC research highlight this disease's complexity, prompting researchers and clinicians to adopt alternative approaches for its treatment. It is important to note that the glycocalyx represents a promising and attractive target for such approaches. The methods applied can be innovative (such as antibodies and nanoparticles) and include well-known drugs previously not used in oncological protocols.

Glycocalyx research, to our opinion, fits into the broader landscape of BC research by offering a novel perspective on tumor biology and potential therapeutic avenues. The glycocalyx plays a crucial role in shaping the tumor microenvironment. Changes in its composition can influence cell–cell interactions, immune responses, and the delivery of drugs and nutrients to the tumor. Understanding these interactions can lead to new strategies for disrupting tumor growth and metastasis [230]. It is involved in various signaling pathways that regulate cell growth, differentiation, and survival. By studying the glycocalyx, researchers can gain insights into how cancer cells communicate with their surroundings and develop strategies to disrupt these communication networks [231]. The glycocalyx can also act as a barrier to drug delivery, limiting the effectiveness of certain therapies. Research on the glycocalyx can help identify ways to overcome this barrier and improve drug delivery to tumor cells. In addition, changes in the glycocalyx have been implicated in the development of drug resistance, making it an important area of investigation for improving cancer treatment outcomes [232, 233]. In addition, alterations in the glycocalyx can serve as potential biomarkers for early detection, prognosis, and treatment response in BC. By analyzing changes in the glycocalyx, researchers may be able to identify patients who are at high risk of developing BC or who are likely to benefit from specific therapies. Taken together, conventional treatments like chemotherapy often face challenges due to the tumor's ability to adapt and evade immune responses. For example, the hypersialylation of the glycocalyx can promote metastasis and resistance to therapies, including HER2-targeted treatments [234]. In addition, dietary interventions targeting glycocalyx sialylation have been explored as a means to modulate metastatic potential, indicating a multifaceted approach to treatment. Overall, glycocalyx targeting may offer a complementary strategy to existing therapies, potentially improving outcomes for patients with BC.

## Data Availability

Data sharing not applicable to this article as no datasets were generated or analyzed during the current study.

## Nomenclature

BC: Breast cancer
CAC: Colitis-associated cancer (a specific type of colorectal cancer that develops in patients with inflammatory bowel disease as a result of prolonged colitis, especially in ulcerative colitis)
CAR: Chimeric antigen receptor (a special receptor created in the laboratory that is designed to bind to certain proteins on cancer cells)
CS: Chondroitin sulfate (a chemical found in human and animal cartilage)
CSPG4: Chondroitin sulfate proteoglycan 4
CTCs: Circulating tumor cells (a cancer cell that breaks away from the original (primary) tumor and enters the bloodstream)
CTGF: Connective tissue growth factor (a key signaling and regulatory molecule involved in different biological processes, such as cell proliferation, angiogenesis, and wound healing, as well as multiple pathologies, such as tumor development and tissue fibrosis)
DRFS: Distant recurrence free survival
DS: Dermatan sulfate (a glycosaminoglycan (formerly called a mucopolysaccharide) found mostly in skin but also in blood vessels, heart valves, tendons, and lungs)
ECM: Extracellular matrix (a large network of proteins and other molecules that surround, support, and give structure to cells and tissues in the body)
EMT: Epithelial-mesenchymal transition (a biologic process that allows a polarized epithelial cell, which normally interacts with basement membrane via its basal surface, to undergo multiple biochemical changes that enable it to assume a mesenchymal cell phenotype)
ER: Estrogen receptor
ETCC1: Electron transport chain complex I
FDA: Food and Drug Administration
GAGs: Glycosaminoglycans
GAGs: Glycosaminoglycans
GPC1: Glypican-1
GPC3-AS1: GPC3 antisense transcript 1
GSLs: Glycosphingolipids
HA: Hyaluronic acid
HASes: Hyaluronan synthases
HBCx: Human BC xenografts
HEPG2: Human liver cancer cell lines
HER2/neu: Human epidermal grows factor receptor 2
HP: Heparin (a naturally occurring glycosaminoglycan)
HPSE1/2: Heparanase-1 /2
HS: Heparan sulfate
HSPG: Heparan sulfate proteoglycan (a large family of macromolecules that are found on the surface of cells and in the surrounding environment)
HSPGs: Heparan sulfate proteoglycans
HYAL1 (2): Hyaluronidase 1 (2) gene
I-CAMs: Intercellular adhesion molecule-1
IKK: I kappa kinase
iNOS: Inducible nitric oxide synthase
Ki67: Marker of proliferation Kiel 67
KS: Keratan sulfate
LMW: Low molecular weight (arbitrarily defined as a molecular weight below 1000 dalton)
LUM: Low lumican
MAbs: Monoclonal antibodies
MAPK: Mitogen-activated protein kinase
miR: microRNA
MRP1: Multidrug resistance protein 1
NEU3: Neurominidarse 3
NK: Natural killer
OS: Overall survival
PDGF-A: Platelet-derived growth factor A
PR: Progesterone receptor
PROTAC: Proteolysis targeting chimeric (a molecule that can remove specific unwanted proteins)
PSGL-1: P-selectin glycoprotein ligand-1
PSGL-1: P-selectin glycoprotein ligand-1
PTM: Posttranslational modification
RECIST: Response evaluation criteria in solid tumors
RHAMM: Receptor for hyaluronan-mediated motility
ROS: Reactive oxygen species
SAL: Salinomycin
SBT-A: Scutebarbatine A
SDC-1: Domain of syndecan-1
SDC-1/4: Syndecan-1/4
Sn: Sialoadhesin (a cell adhesion molecule found on the surface of macrophages)
sTN: Sialyl-Tn
TF: Tensile force
TME: Tumor microenvironment
TNBC: Triple-negative breast cancer
UTR: Untranslated region
ZA: Zoledronic acid
